# Thymomas: a cytological and immunohistochemical study, with emphasis on lymphoid and neuroendocrine markers

**DOI:** 10.1186/1746-1596-2-13

**Published:** 2007-05-11

**Authors:** Borislav A Alexiev, Cinthia B Drachenberg, Allen P Burke

**Affiliations:** 1 Department of Pathology, University of Maryland Medical Center, 22 S Greene Street, Baltimore, MD 21201, USA

## Abstract

**Background:**

The current study correlates cytologic morphology with histologic type and describes immunophenotypes with a focus on epithelial, neuroendocrine, and lymphoid characteristics in an institutional series of surgically excised thymomas.

**Methods:**

Fine needle aspirates (FNAs) and surgical specimens were retrospectively analyzed, and immunohistochemical stains were performed for EMA, cytokeratin 7, cytokeratin 20, CD57 CD5, bcl-2, calretinin, vimentin, CD3, CD20, CD1a, CD99 and Ki67. Tumors were classified by WHO criteria.

**Results:**

There were eleven male and six female patients with an age range of 41 to 84 years (mean, 61 years) and a male to female ratio of 1.8:1. Four thymomas (4/17, 23.5%) were associated with neuromuscular disease: myasthenia gravis (n = 3) and limbic encephalitis (n = 1). FNA, under CT guidance, was performed in 7 cases. The positive predictive value for thymoma by FNA cytology was 100% and the sensitivity was 71%. Thymomas associated with neuromuscular disorders were WHO types B2 (n = 1) and B3 (n = 3), and showed a strong expression of CD57 in the majority of neoplastic epithelial cells accompanied by large numbers of CD20+ intratumoral B lymphocytes. Two of seventeen (11.7%) thymomas (all sporadic B3 type) contained numerous neoplastic epithelial cells positive for CD5 and bcl-2.

**Conclusion:**

Our results suggest that thymomas associated with autoimmune disorders contain a significant population of CD20+ intratumoral B lymphocytes. Strong CD57 positivity in thymomas may suggest a concomitant neuromuscular disorder, notably myasthenia gravis. CD5 expression is of limited value in the differential diagnosis of primary thymic epithelial neoplasms since both thymic carcinomas and thymomas may express CD5.

## Background

Thymoma is a primary mediastinal neoplasm arising from or exhibiting differentiation towards thymic epithelial cells, typically with the presence of non-neoplastic lymphocytes [1]. The overall incidence of thymoma in the US is 0.15 per 100,000 person-years [2]. The cause of thymoma is unknown [3]. The histological classification of thymoma has remained controversial since 1999, when the World Health Organization (WHO) Consensus Committee published a histologic typing system for tumors of the thymus [4]. The 2004 WHO Classification of Lung, Pleura, Thymus and Heart Tumors [1] maintained the skeleton of the 1999 classification with few additional changes. Numerous subsequent studies demonstrated that the WHO histologic classification is a significant independent prognostic factor [5-10]. The cytologic diagnosis of thymomas is challenging. In part, this is because the neoplasm is uncommon and aspirates are infrequently encountered [11]. The immunohistochemical profile of thymomas is complex due to the variety of growth patterns and background lymphoid infiltrate. We studied a series of thymomas a) to correlate cytologic morphology with the current WHO classification scheme, and b) to further delineate type-specific immunophenotypes with a focus on epithelial, neuroendocrine, and lymphoid characteristics.

## Methods

Data were evaluated retrospectively from patients who underwent fine needle aspiration (FNA) cytology (n = 7) and surgical treatment (n = 17) for thymoma at the University of Maryland Medical Center between 2000 and 2006. For cytologic examination utilizing CT guidance, 2–3 FNAs using a 20 gauge Franseen needle were performed. Air dried smears were prepared and stained with Diff Quick for immediate cytological evaluation. After each pass the needle was rinsed in RPMI medium. The rinsings were cytocentrifuged and resulting smears were immediately fixed in 95% alcohol and stained by the Papanicolaou method. Hematoxylin-eosin stained cell block sections were available in all cases examined. Tissue samples from the surgical specimens were fixed in 10% formaldehyde and embedded in paraffin. Five-micron tissue sections were stained with hematoxylin-eosin. The tumors were classified according to the World Health Organization criteria [1]. The scheme developed by Masaoka [12] as modified by Koga et al. [13] was used for staging.

### Immunohistochemistry

Immunohistochemical staining was performed using an automated slide preparation system (Benchmark XT, Ventana, Tuscon, AZ), a Ventana Biotin-Streptavidin (B-SA) Enhanced DAB Detection Kit (Ventana, Tucson, AZ), and commercially available prediluted monoclonal antibodies: CD3, CD5, CD20, CD1a, CD99, calretinin, vimentin, CK7, CK20, Epithelial Membrane Antigen (EMA), AE1/AE3, CD57, Ki67, and bcl-2 (all Ventana, Tucson, AZ).

## Results

### Demographic and clinical data

There were eleven male and six female patients with an age range of 41 to 84 years (mean, 61 years) and a male to female ratio of 1.8:1. Four thymomas (4/17, 23.5%) were associated with neuromuscular disease: myasthenia gravis (n = 3) and limbic encephalitis (n = 1). All thymomas associated with myasthenia gravis were type B3, whereas limbic encephalitis occurred in a patient with type B2 thymoma.

### Cytologic findings

**Figure 1:**
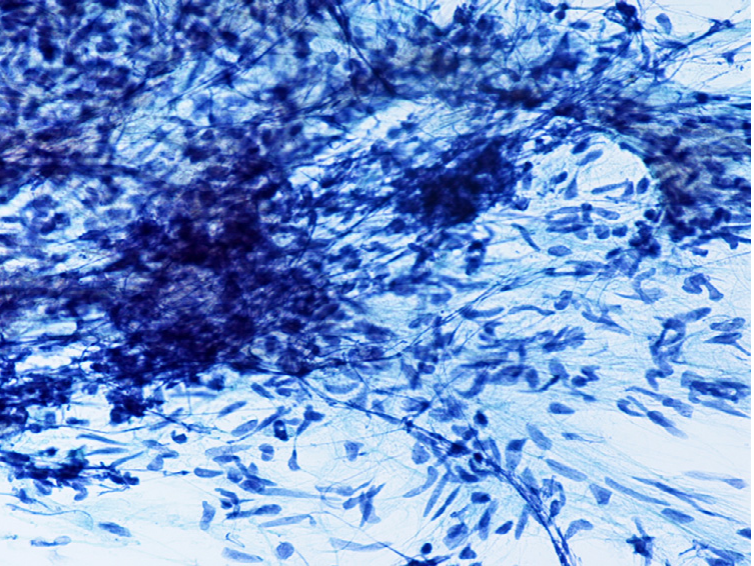
(Papanicolaou stain, × 200): Type AB thymoma. FNA smear shows elongate neoplastic epithelial cells with oval or spindle pale nuclei and inconspicuous nucleoli.

**Figure 2:**
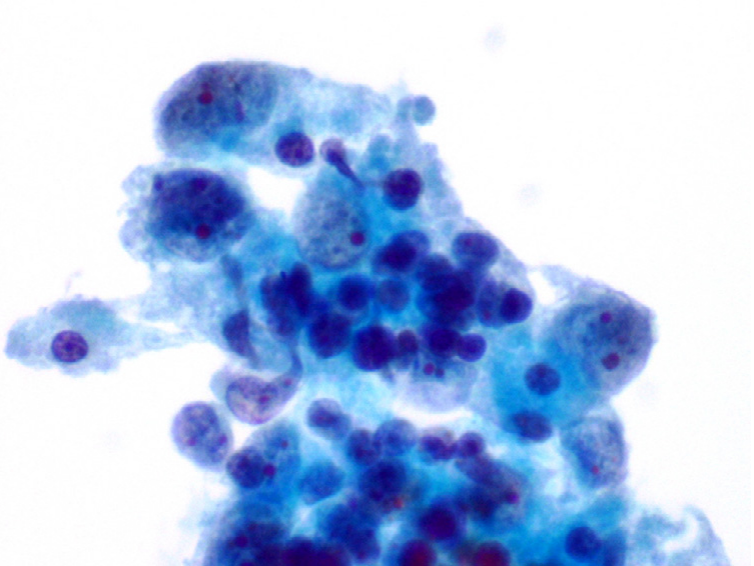
(Papanicolaou stain, × 600): Type AB thymoma. FNA smear shows neoplastic epithelial cells with dispersed chromatin and small nucleoli, and background lymphocytes.

Fine needle aspirates (FNAs) were moderately cellular. Tissue fragments of variable size were frequently observed. The aspirates consisted of a dual population of oval or elongate epithelial cells and mature-appearing lymphocytes. The neoplastic epithelial cells contained round, oval or spindle pale nuclei with dispersed chromatin and inconspicuous nucleoli (Figs. 1 and 2). The cytoplasm was scant, and the cell borders were ill-defined. The nuclear/cytoplasmic ratio was moderate to high. Nuclear pleomorphism was minimal; mitoses were not seen. In two cases, the epithelial cells were obscured by a predominant lymphocytic infiltration. Immunocytochemical staining of cell block sections with pan-cytokeratin (AE1/AE3) revealed the epithelial nature of the neoplastic cells. Correlation of cytological diagnosis by FNA cytology with the final histological diagnosis is shown in table 1. In two cases, the aspirates were non-diagnostic. The positive predictive value for thymoma by FNA cytology was 100% while the sensitivity was 71%.

**Table 1:** Correlation of Cytological Diagnosis with Final Histological Diagnosis

Case	Cytological Diagnosis	Histological Diagnosis
1	Non-diagnostic	Type B3 thymoma
2	Thymoma	Type AB thymoma
3	Non-diagnostic	Type B3 thymoma
4	Thymoma	Type AB thymoma
5	Thymoma	Type B1 thymoma
6	Thymoma	Type A thymoma
7	Thymoma	Type B1 thymoma

### Histologic and immunohistochemical findings

The pathological diagnoses, tumor stages, and immunohistochemical findings are presented in tables 2, 3, 4.

**Table 2:** Pathologic Diagnosis and Thymoma Pathologic Staging*

Thymoma type	Stage I	Stage IIa	Stage IIb	Stage III	Stage IVa
A (n = 2)	1	1	-	-	-
AB (n = 3)	1	2	-	-	-
B1 (n = 4)	1	2	1	-	-
B2 (n = 1)	-	1	-	-	-
B3 (n = 6)	2	2	-	1	1
MNT (n = 1)	1	-	-	-	-

* = references 12 and 15

MNT = micronodular thymoma

**Table 3:** Immunohistochemical Results, Tumor Cell Expression

Marker	Thymoma Type					
	
	A	AB	B1	B2	B3	MNT
EMA	+	+	Focal	S	+	+
CK7	+	+^	+	+	+	+
CK20	-	-	-	-	-	-
CD57	S	S	S	+*	+*	+
CD5	-	-	-	-	-/+	-
Bcl-2	-	-	-	-	-/+	-
Calretinin	S	S	S	S	S	S
Vimentin	+	+	-	-	-	-
CD20	+	+	-	-	-	-

MNT= micronodular thymoma

S = single cells

+^ = neoplastic epithelial cells in type B component were CK7 negative

-/+ = variably (2 sporadic type B3 thymomas stained strongly positive)

+* = strong expression in type B2 (n = 1) and type B3 (n = 3) thymomas associated with autoimmune disorder; scattered positive neoplastic cells in sporadic thymomas

**Table 4:** Immunohistochemical Results, Background Lymphocytes

Marker	Thymoma Type					
	
	A	AB	B1	B2	B3	MNT
CD57	S	S	S	S	S	S
CD3	S	+	+	+	+	+
CD5	S	+	+	+	+	+
CD20	-	+	+	-/+	-/+	+*
CD1a	-	+	+	+	+	+
CD99	-	+	+	+	+	+
Ki67/MIB1	<10%	>80%	>80%	>80%	>80%	>80%

MNT = multinodular thymoma

-/+ = variably (large numbers of intratumoral CD20+ lymphocytes in type B2 (n = 1) and type B3 (n = 3) thymomas associated with autoimmune disorder)

* = numerous large stromal CD20+ lymphoid aggregates

S = single cells

In type A thymoma, neoplastic epithelial cells stained strongly for EMA, CK7, vimentin, and CD20. Immunostaining for CD57 and calretinin was noted in scattered neoplastic epithelial cells. CK20, CD3, CD5, bcl-2, CD99, and CD1a were all negative in neoplastic epithelium. The few lymphocytes were CD3+ and CD5+, and CD20-, CD1a- and CD99-. The Ki67 proliferation index was low in neoplastic epithelial cells (<10%).

**Figure 3:**
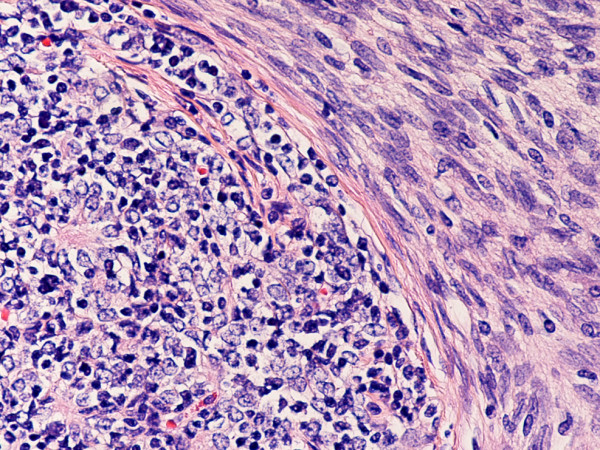
(hematoxylin-eosin, × 200): Type AB thymoma. Note type A and type B components. (hematoxylin-eosin, × 200)

**Figure 4:**
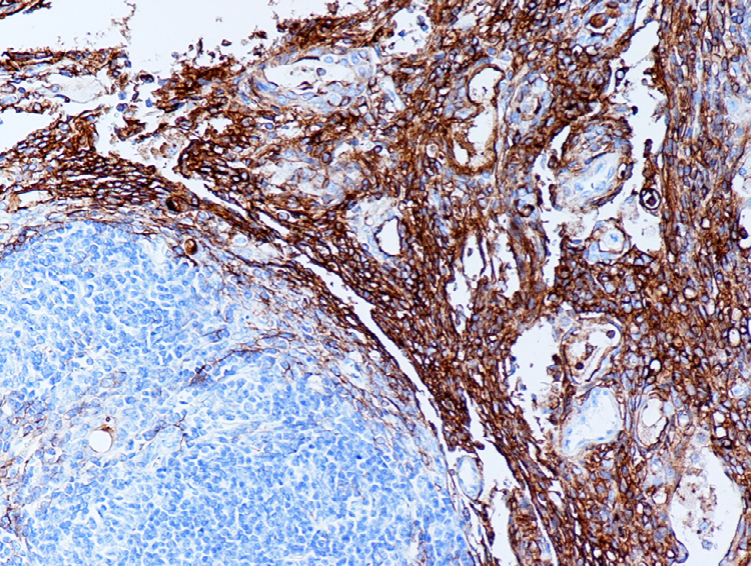
(B-SA, anti-EMA, × 200): Type AB thymoma. Neoplastic epithelial cells in type A component express EMA.

**Figure 5:**
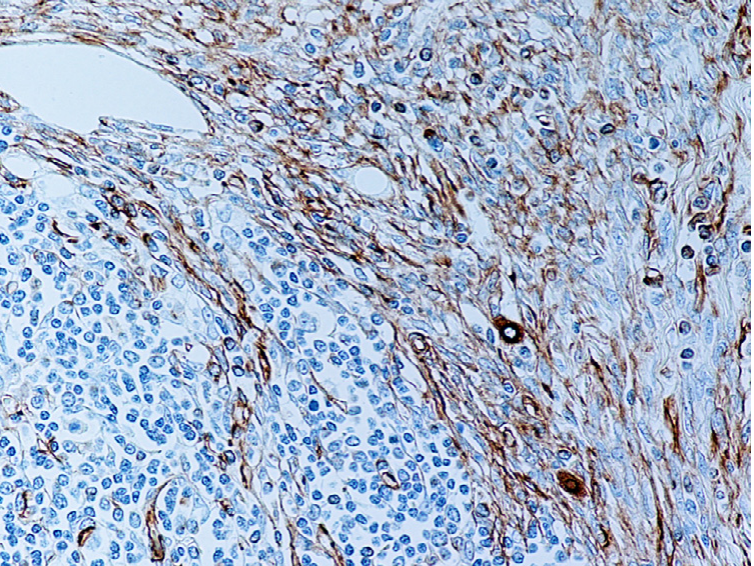
(B-SA, anti-vimentin, × 200): Type AB thymoma. Neoplastic epithelial cells in type A component express vimentin.

**Figure 6:**
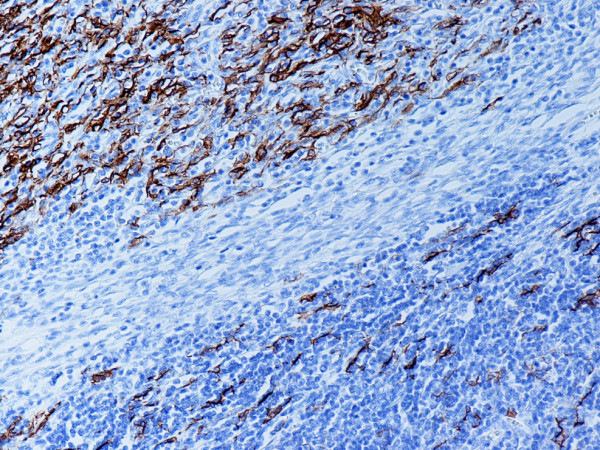
(B-SA, anti-CD20, × 200): Type AB thymoma. Neoplastic epithelial cells in type A and B components express CD20.

The type A component in AB thymomas showed an immunophenotype virtually indistinguishable from the one of type A neoplasms, including the strong expression of vimentin (Figs. 3, 4, 5, 6). In the type B component, the neoplastic epithelial cells were CK7-and variably EMA+, vimentin +, and CD20+. Focal weak CD57 positivity was also observed. The majority of non-neoplastic lymphocytes showed an immunophenotype characteristic of immature T cells (CD3+, CD5+, CD1a+, CD99+; Ki67 proliferation index > 80%). Single CD57+ cytotoxic T lymphocytes were rarely seen. The lymphocytes in foci of medullary differentiation showed a mature T cell phenotype (CD3+, CD5+, CD1a-, CD99-; Ki67 proliferation index <10%) and contained a large number of CD20+ B lymphocytes.

In type B1 thymomas, neoplastic epithelial cells showed expression of EMA and CK7 at the stromal interface, and were consistently CD20, CK20, CD5, and vimentin negative. There were rare cells positive for CD57 and calretinin. Admixed T lymphocytes were CD3+, CD5+, CD1a+, CD99+, with high Ki67 proliferation index (>80%), whereas lymphocytes in the medullary islands were mature T cells (CD3+, CD5+, CD1a-, CD99-; Ki67 proliferation index <10%) and CD20+ B cells. Few CD57+ cytotoxic T lymphocytes were also noted.

**Figure 7:**
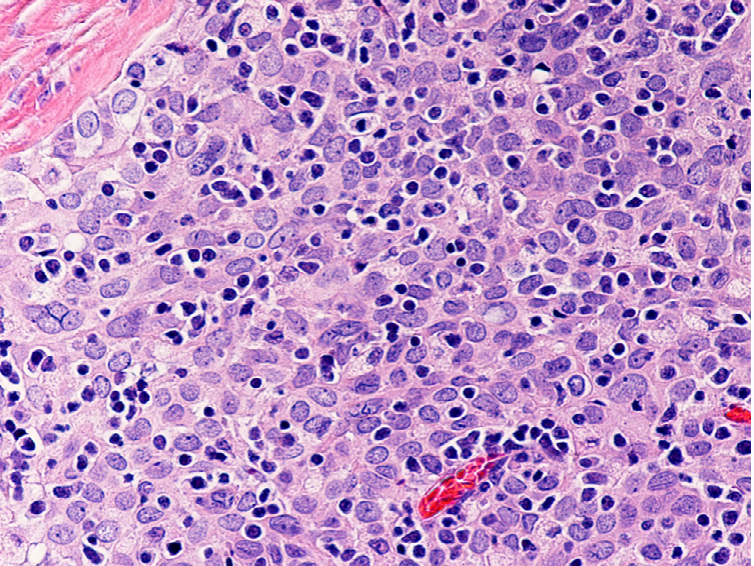
(B-SA, anti-CD20, × 200): Type B3 thymoma associated with myasthenia gravis.

**Figure 8:**
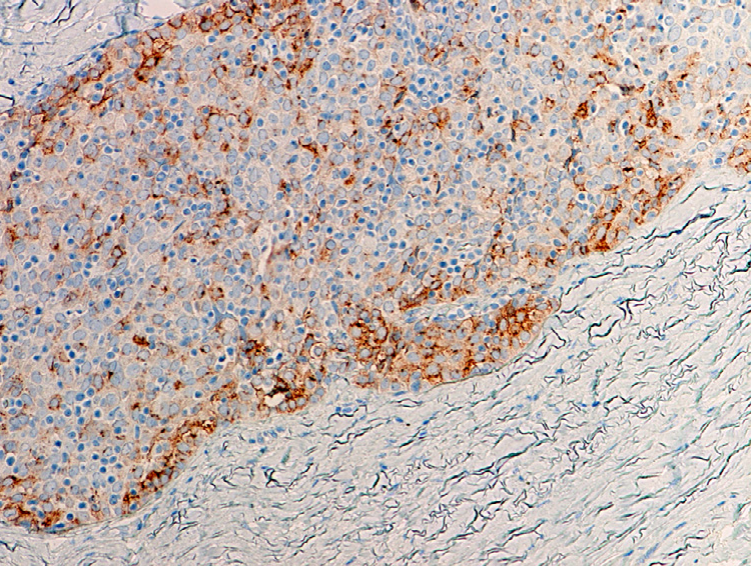
(B-SA, anti-CD57, × 200):Type B3 thymoma associated with myasthenia gravis. Strong expression of CD57 in the majority of neoplastic cells.

**Figure 9:**
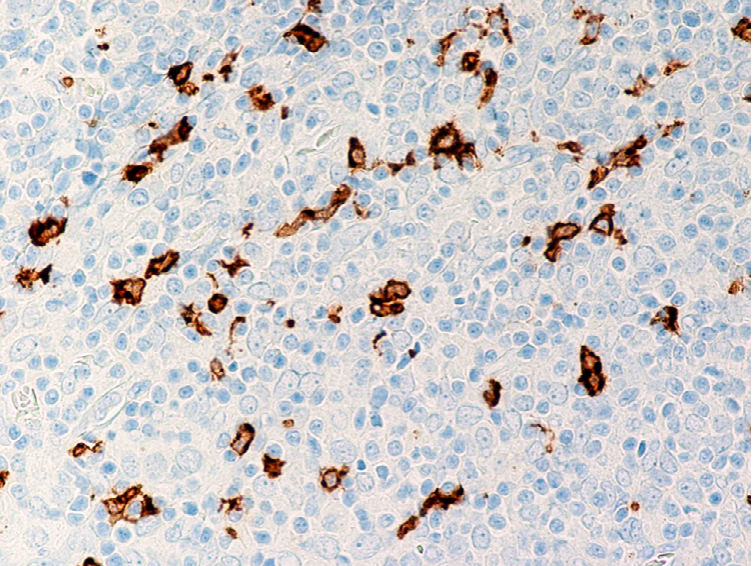
(B-SA, anti-CD20, × 200): Type B3 thymoma associated with myasthenia gravis. Intratumoral lymphocytes and single fibroblast-like cells express CD20.

**Figure 10:**
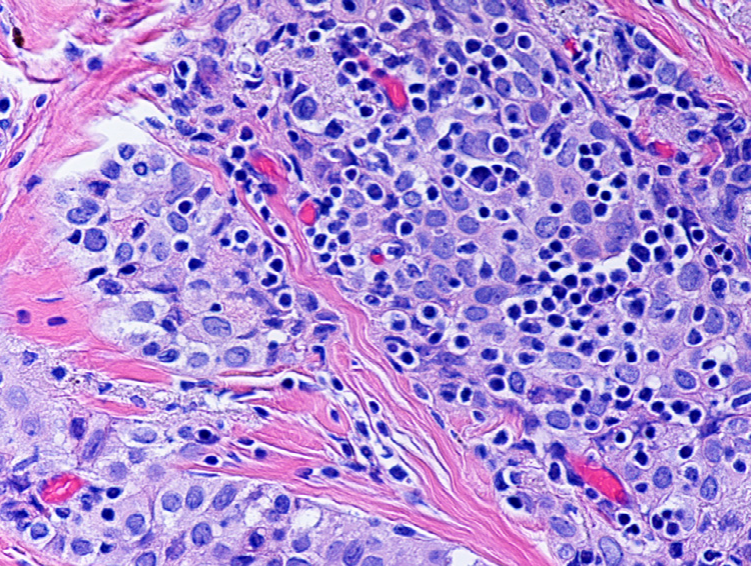
(hematoxylin-eosin, × 400): Type B3 thymoma not associated with autoimmune disease.

**Figure 11:**
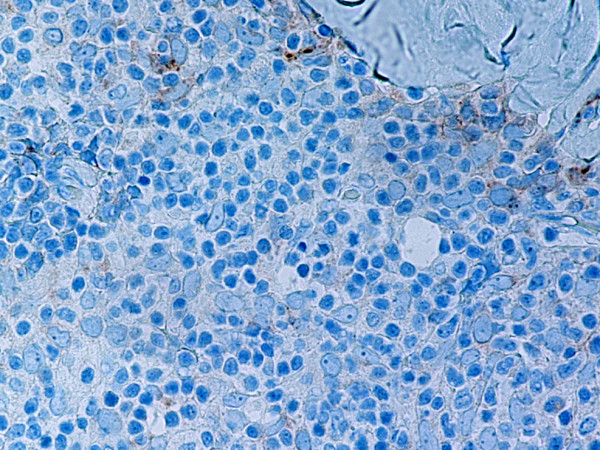
(B-SA, anti-CD57, × 400): Type B3 thymoma not associated with autoimmune disease. Single neoplastic epithelial cells express CD57.

**Figure 12:**
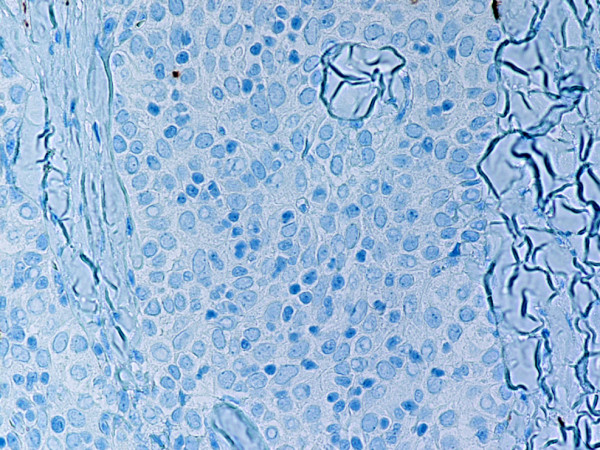
(B-SA, anti-CD20, × 400): Type B3 thymoma not associated with autoimmune disease. No CD20+ intratumoral lymphocytes are seen.

**Figure 13:**
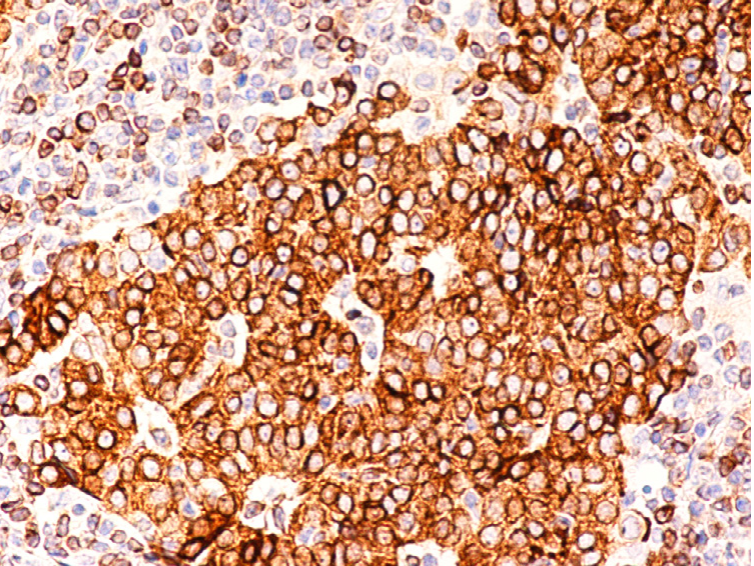
(B-SA, anti-CD57, × 400): Type B3 thymoma. Note strong expression of CD5 in neoplastic epithelial cells and background lymphocytes.

**Figure 14:**
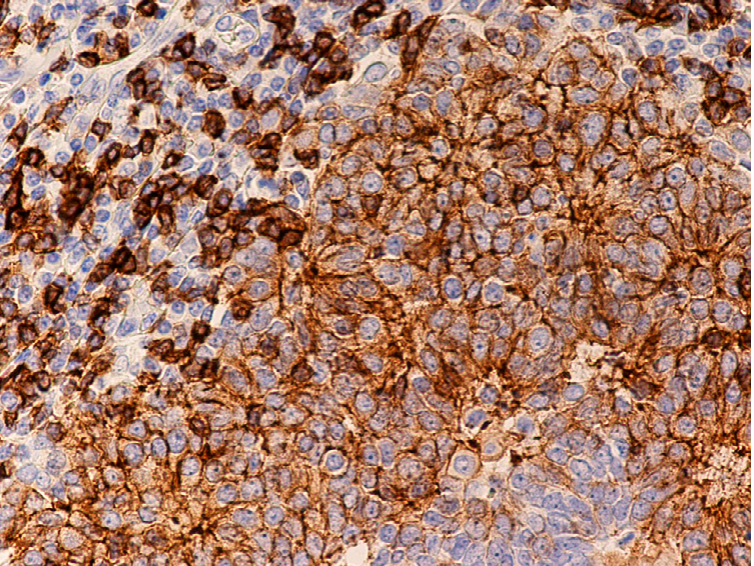
(B-SA, anti-bcl-2, × 400): Type B3 thymoma. Note strong expression of bcl-2 in neoplastic epithelial cells and background lymphocytes.

Type B2 and B3 thymomas showed a similar immunophenotype with a variable CK7 expression, and immature intratumoral non-neoplastic T lymphocytes (CD3+, CD5+, CD1a+, CD99+; Ki67 proliferation index >80%). In type B2 thymomas, EMA was expressed in single neoplastic cells, whereas type B3 thymomas demonstrated an expression of EMA at the tumor/stroma interface and adjacent to cystic spaces. Small stromal lymphoid aggregates containing mature T lymphocytes (CD3+, CD5+, CD1a-, CD99-, Ki67 proliferation index <10%) and CD20+ B lymphocytes were observed in all B2 and B3 thymomas. Scattered CD57+ cytotoxic T lymphocytes were also noted. Vimentin was not expressed by neoplastic epithelial cells. An unexpected observation was the diffuse strong expression of CD57 in the majority of neoplastic epithelial cells, large numbers of CD20+ intratumoral lymphocytes and single CD20+ elongate fibroblast-like cells in all thymomas associated with neuromuscular disorders (Figs. 7, 8, 9). In contrast, sporadic thymomas contained only few scattered CD57+ neoplastic epithelial cells and CD20+ B cells (Figs. 10, 11, 12). Two of seventeen (11.7%) thymomas (all sporadic type B3) contained numerous CD5+, bcl-2+ neoplastic epithelial cells (Figs. 13 and 14).

**Figure 15:**
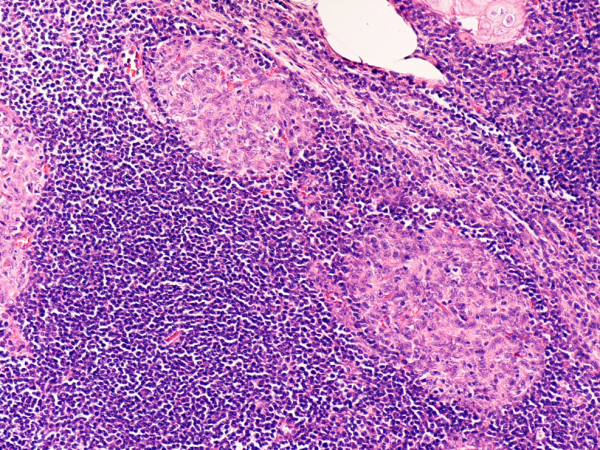
(hematoxylin-eosin, × 100): Micronodular thymoma.

**Figure 16:**
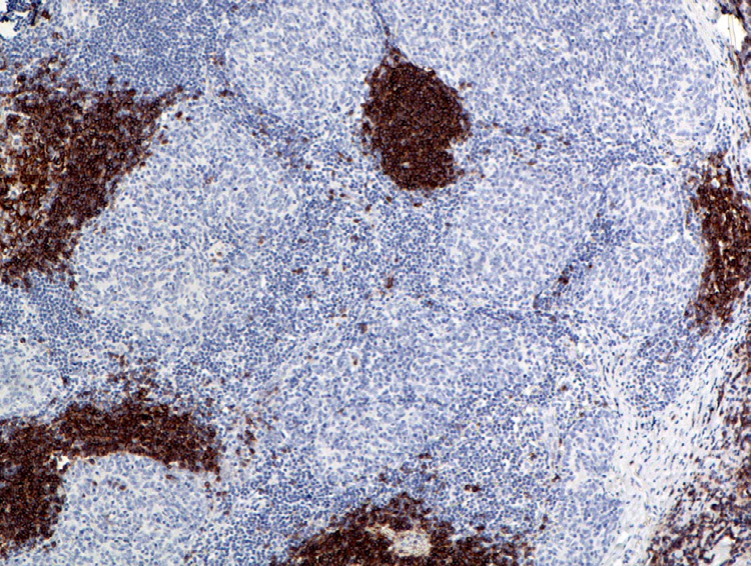
(B-SA, anti-CD20, × 200): Micronodular thymoma. Note large lymphoid aggregates positive for CD20.

**Figure 17:**
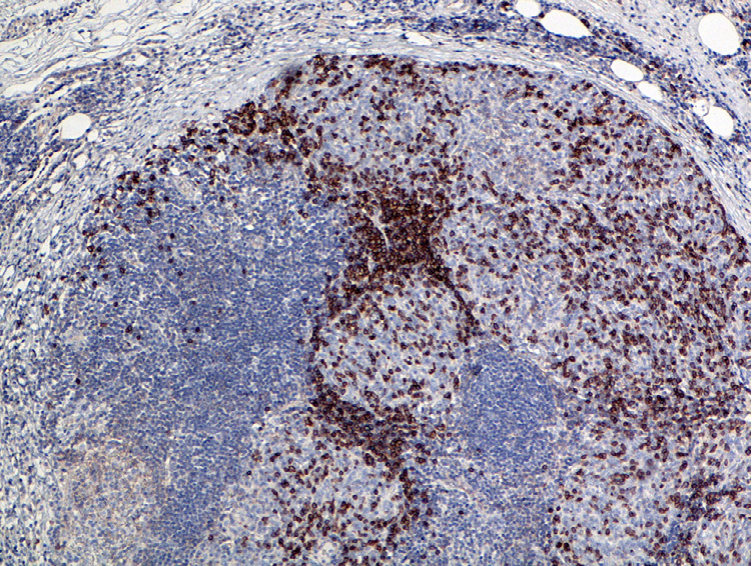
(B-SA, anti-CD99, × 200): Micronodular thymoma. Note strong expression of CD99 in intratumoral lymphocytes.

**Figure 18:**
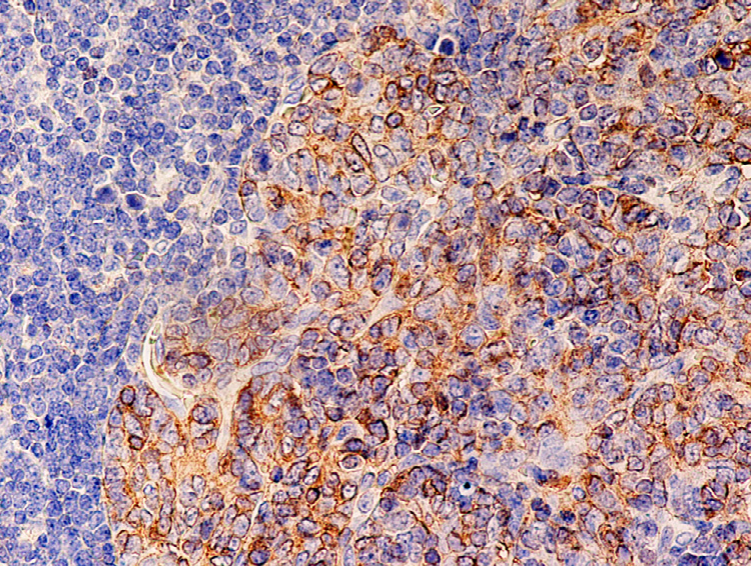
(B-SA, anti-CD57, × 400): Micronodular thymoma. Neoplastic epithelial cells express CD57.

The sporadic micronodular thymoma (MNT) showed a unique immunophenotype with neoplastic epithelial cells strongly positive for EMA, CD57 (unlike other sporadic neoplasms), and focally for CK7 (Figs. 15, 16, 17, 18). CK20, CD20, vimentin, and CD5 were all negative. The stroma contained large lymphocytic aggregates containing a predominant cell population of CD20 B lymphocytes and mature T cells (CD3+, CD5+, CD1a-, CD99-, Ki67 proliferation index <10%). Intratumoral non-neoplastic immature T lymphocytes were numerous and expressed CD3, CD5, CD1a, and CD99, with high Ki67 proliferation index (>80%).

## Discussion

Thymoma is one of the major differential diagnostic considerations in an adult patient > 40 years of age presenting with a mediastinal mass. Aspirates of thymomas are distinguishable from other lesions, and FNA and CT-guided core needle biopsy are proven methods for investigating mediastinal masses [14]. The cytologic diagnosis of thymoma can be extremely challenging. In part, this is because a technically proficient interventional radiologist is needed, epithelial cells may be difficult to recognize in lymphoid rich aspirate smears, and there is inherent sampling error in a tumor that frequently displays heterogeneous histopathology [11]. In the present study the positive predictive value for thymoma by FNA cytology was 100% and sensitivity – 71%. Previous observations showed a diagnostic sensitivity of 86% for FNAs [15] and 100% for ultrasonically guided core needle biopsy [3], respectively. Thymoma should be differentiated from other anterior mediastinal neoplasms with epithelial and/or lymphoid differentiation, including Non-Hodgkin (NHL) and Hodgkin lymphomas, thymic carcinomas, and germ cell malignancies. NHL and Hodgkin lymphoma can be separated from thymoma by their dispersed cell population, distinctive cytologic features, and positive staining for CD45, CD20, CD15, and CD30, respectively. Helpful cytologic and immunocytochemical features in making the diagnosis of thymic carcinoma are clear-cut cytological atypia, absence of immature lymphocytes (CD1a+, CD99+), and expression of CD5 and CD70 by neoplastic epithelial cells [16]. Mediastinal seminomas are immunoreactive for PLAP and CD117, while CD30 is expressed in 85–100% of embryonal carcinomas [1]. Similar to thymomas, mediastinal neuroendocrine neoplasms express CD57 and other neuroendocrine markers but are consistently negative for non-neoplastic immature lymphocytes (CD1a+, CD99+). Limitations of the cytological method include an unproven ability to definitively separate thymoma into specific WHO subtypes using cytology alone, and to determine capsular invasion [11]. Overall, the present study confirms previous observations [15,16] that FNA of anterior mediastinal thymic lesions generally yields adequate cellular tissue with distinct cytologic and immunophenotypic features that enables thymoma diagnosis.

Thymomas can exhibit a spectrum of autoimmune phenomena, comprising neuromuscular, hematopoietic, dermatologic, rheumatic/vasculitic, hepatic and renal diseases [1]. In our study 4/17 (23.5%) thymomas were associated with myasthenia gravis (n = 3) or limbic encephalitis (n = 1). All thymomas with myasthenia gravis were type B3 thymoma, whereas limbic encephalitis occurred in a patient with type B2 thymoma. All four neoplasms displayed a diffuse strong expression of the neuroendocrine marker CD57. In contrast, thymomas not associated with neuromuscular disorders contained only few scattered CD57+ neoplastic epithelial cells. Only few studies have addressed the prevalence of neuroendocrine differentiation in human thymic neoplasms and demonstrated reactivity for synaptophysin, neuron-specific enolase, and/or chromogranin in 58% thymic carcinomas and atypical thymomas [17-20]. In addition, myasthenia gravis was present in 2/6 thymus neoplasms with neuroendocrine differentiation [17]. Intriguingly, our cases were dissimilar to those described in earlier reports in terms of the CD57 positivity in type A and type AB thymomas [21]. In their excellent study, Pan et al [21] reported CD57 positivity in 76% short-spindled and 80% long-spindled variants. Unfortunately, the CD57 staining was not correlated with the presence or absence of autoimmune disease in thymoma patients. In this study, CD57 expression was seen only in scattered neoplastic epithelial cells in type A thymomas. Focal weak CD57 positivity in type AB thymomas was also noted. None of the type A and type AB neoplasms were associated with a neuromuscular disorder. This discrepancy cannot be explained at the present time. Whether and in what percentage of cases immunohistochemical reactivity for CD57 may be correlated with clinical behavior and outcome remains a controversial issue. The results of this study suggest that a diffuse strong CD57 expression by neoplastic epithelial cells has a high correlation with the concomitant presence of a neuromuscular disease, notably myasthenia gravis.

The biologic significance of CD20 positivity in background lymphocytes remains unknown. As the data in this study show, large numbers of intratumoral CD20+ B lymphocytes were found in all 4 cases of thymoma associated with neuromuscular disorders, whereas sporadic thymomas contained only few scattered intratumoral CD20+ B cells. Numerous CD20+ B lymphocytes were also observed in the medullary islands in type AB and type B1, and in stromal lymphoid aggregates in type B2 and B3 thymomas.

In this context, Fend et al. [22] reported that large numbers of B cells with germinal centre formation were found almost exclusively in myasthenia gravis-associated tumors, mainly in cortical thymomas. As far as the occurrence of B-cell differentiation in medullary islands is concerned, it has been suggested that a medullary microenvironment with epidermoid cells corresponding to Hassall's corpuscles seems to be necessary for specific intrathymic B cell homing [22].

Surprisingly, we observed in type A and type AB thymomas a large number of CD20+, EMA+, vimentin+ fibroblast-like elongate neoplastic epithelial cells. In type AB neoplasms, the majority of positive cells were located in the type A component and, to a lesser degree, in the type B component, in the neoplastic epithelial meshwork. No CD20+, vimentin+ neoplastic epithelial cells were seen in type B thymomas. These data are in agreement with the observed variable numbers of CD20+ spindle epithelial cells in mixed thymomas [23] and suggest that neoplastic epithelial cells in type A and type AB thymomas are distinctive and different from either B1, B2, B3 thymoma.

MNT is a rare neoplasm, characterized by a micronodular growth pattern associated with florid lymphoid follicular hyperplasia of the stroma [24]. In agreement with previous reports [24-26], in this study, the stromal lymphoid cell population in the MNT contained numerous CD20+ B cells and mature T cells, whereas intratumoral lymphocytes showed an immature T cell phenotype (CD3+, CD1a+, CD99+, Ki67 proliferation index >80%). The biologic significance of CD57 expression by neoplastic epithelial cells in MNT is unknown. Unlike the type B2 and B3 thymomas associated with neuromuscular disorders, the MNT in this study did not contain CD20+ intratumoral lymphocytes. The patient had no clinical signs or history of myasthenia gravis. Earlier studies have shown that MNT is not associated with autoimmune disease [24]. Recent findings suggest that expression of chemoattractants, such as CCL18, CCR6, and CCL20, on neoplastic epithelium in MNT can promote the recruitment of MALT, the emergence of monoclonal B cells, and, eventually, the subsequent development of mediastinal lymphoma [25].

Two of seventeen (11.7%) thymomas (all sporadic B3 type) contained numerous CD5+ and bcl-2+ neoplastic epithelial cells. Previous studies showed that epithelial cells in B3 thymomas are negative for CD5 [1]. The data suggest that CD5 is not a reliable diagnostic marker for primary thymic carcinomas since CD5 may be expressed also in thymomas. The biologic significance of bcl-2 expression in neoplastic epithelium is unknown. Previous reports suggested that bcl-2 acts as an inhibitor of apoptosis in thymomas and correlates with aggressiveness in thymic epithelial neoplasms [4,27,28].

## Conclusion

In conclusion, the present report expands the spectrum of our knowledge showing that thymomas associated with autoimmune disorders contain a large population of CD20+ intratumoral B lymphocytes. Strong CD57 positivity in neoplastic epithelial cells in thymomas may suggest a concomitant neuromuscular disorder, notably myasthenia gravis. Immunocytochemical analysis is useful in assisting in the identification of these neoplasms and in excluding other malignancies in CT-FNA. Limitations of the cytological method include an inability to definitively separate thymoma into specific WHO subtypes using cytology alone, and to determine capsular invasion. CD5 expression is of limited value in the differential diagnosis of primary thymic epithelial neoplasms since both thymic carcinomas and thymomas may express CD5.

## Competing interests

The author(s) declare that they have no competing interests.

## Authors' contributions

BAA evaluated the FNAs and immunohistochemical stains, confirmed the diagnosis, designed the report and drafted the manuscript. CID and APB provided consultation.

All authors read and approved the final manuscript.
